# Sudarshan Kriya Yoga Breathing and a Meditation Program for Burnout Among Physicians: A Randomized Clinical Trial

**DOI:** 10.1001/jamanetworkopen.2023.53978

**Published:** 2024-01-31

**Authors:** Asli Korkmaz, Guro Pauck Bernhardsen, Burcu Cirit, Gayem Koprucu Suzer, Hale Kayan, Hülya Biçmen, Muratcan Tahra, Asli Suner, Soili Marianne Lehto, Duygu Sag, Fahri Saatcioglu

**Affiliations:** 1 Department of Genomic Sciences and Molecular Biotechnology, Izmir International Biomedicine and Genome Institute, Dokuz Eylul University, Izmir, Turkey; 2 Izmir Biomedicine and Genome Center, Izmir, Turkey; 3 Division of Mental Health Services, Department of Research and Development, Akershus University Hospital, Lørenskog, Norway; 4 University of Health Sciences, Ataturk Chest Diseases Education and Research Hospital, Ankara, Turkey; 5 American Hospital and Koç University Hospital, Istanbul, Turkey; 6 Manisa Dialysis Center, Manisa, Turkey; 7 Bati Medical Center, Didim, Aydin, Turkey; 8 School of Medicine, Dokuz Eylul University, Izmir, Turkey; 9 Department of Biostatistics and Medical Informatics, Faculty of Medicine, Ege University, Izmir, Turkey; 10 Institute of Clinical Medicine, University of Oslo, Oslo, Norway; 11 Department of Psychiatry, University of Helsinki, Helsinki, Finland; 12 Department of Medical Biology, School of Medicine, Dokuz Eylul University, Izmir, Turkey; 13 Department of Biosciences, University of Oslo, Oslo, Norway; 14 Institute for Cancer Genetics and Informatics, Oslo University Hospital, Oslo, Norway

## Abstract

**Importance:**

Physicians are exposed to high stress and strain that results in burnout, which affects them, their families, their patients, and the entire health care system; thus, there is an urgent need to develop methods to increase the resiliency of physicians. Sudarshan Kriya Yoga (SKY) is a comprehensive yoga breathing and meditation-based program that is a potential approach to mitigate physician burnout.

**Objective:**

To determine whether SKY can reduce psychological distress and improve wellness in physicians.

**Design, Setting, and Participants:**

This randomized clinical trial assessed the potential efficacy of SKY compared with a stress management education (SME) training as control. This study was conducted online from November 11, 2021, to March 14, 2022, and included physicians from Turkey, Germany, and Dubai. Both the SKY and the SME control groups received 1.5 hours of training for 3 consecutive days via a group video conference call. Participants were physicians willing to do some form of relaxation exercise everyday for 2 months. Exclusion criteria included presence of major illness and maintaining a regular mind-body program practice. Statistical analysis took place from March to November 2023.

**Interventions:**

Participants were randomly assigned 1:1 into 2 groups—the SKY group or the SME (control) group—using a computer algorithm. After the 3-day instruction period, the participants in the SKY group practiced for approximately 30 minutes per day on their own and participated in a weekly 1-hour, group-based online follow-up practice. After the 3-day instruction period, participants in the SME group reviewed and applied the notes from stress management education training at their initiative and had a weekly 1-hour group-based online follow-up session.

**Main Outcomes and Measures:**

The primary outcomes were stress and depression (measured by the 42-item Depression, Anxiety, and Stress Scale [DASS-42]) and insomnia measured by the Regensburg Insomnia Scale (RIS) with primary end point at 8 weeks. Secondary outcomes included anxiety (DASS-42); optimism (Life Orientation Test-Revised [LOT-R]); professional fulfillment, work exhaustion, interpersonal disengagement, and overall burnout (Professional Fulfillment Index [PFI]); and self-reported professional errors (Self-Reported Professional Error Questionnaire).

**Results:**

This study included 129 participants (SME, 63 participants [48.9%]; SKY, 66 participants [51.1%]; 115 females [89.2%]; 14 males [10.8%]; mean [SD] age, 46.2 [9.0] years). Compared with the SME control group, participants in the SKY group had significantly decreased stress on the DASS-42 at posttraining (difference, −6.8 points; 95% CI, −9.6 to −4.1 points; *P* = .006) and at postintervention (difference, −6.0 points; 95% CI, −8.8 to −3.3 points; *P* = .03), significantly decreased depression at posttraining (difference, −5.7 points; 95% CI, −8.6 to −2.8 points; *P* < .001) and postintervention (difference, −5.4 points; 95% CI, −8.3 to −2.5 points; *P* < .001), and significantly decreased anxiety at postintervention. In addition, there was a significant decrease in insomnia from baseline to postintervention in the SKY group (difference, −0.3 points; 95% CI, −2.3 to 1.7 points; *P* = .01). The SKY group also showed significantly increased professional fulfillment as well as significant decreases in work exhaustion, interpersonal disengagement, and burnout. There was no effect on self-reported medical errors.

**Conclusions and Relevance:**

In this randomized clinical trial, physicians who regularly practiced SKY throughout a 2-month period experienced improvements in wellness and decreased burnout. These data suggest that SKY may be an effective, practical, and safe strategy to increase wellness and mitigate burnout in physicians.

**Trial Registration:**

ClinicalTrials.gov Identifier: NCT05956470

## Key Points

**Question:**

Does Sudarshan Kriya Yoga (SKY) practice reduce stress and increase well-being in practicing physicians?

**Findings:**

This randomized clinical trial of 129 practicing physicians found that SKY significantly reduced stress, anxiety, and depression on the 42-Item Depression, Anxiety, and Stress scale by 4 to 6 points. In addition, insomnia decreased on the Regensburg Insomnia Scale by 3 points; all differences were statistically significant compared with the control group.

**Meaning:**

These findings suggest that SKY represents a practical and efficient approach for improving physician well-being.

## Teaser

This randomized clinical trial assesses the potential efficacy of Sudarshan Kriya Yoga to reduce psychological distress and improve wellness in physicians compared with stress management education.

## Introduction

The high degree of stress and burnout among physicians and other health care workers has been well established, with a high percentage of physicians experiencing burnout.^1,2,3,4^ This has serious adverse effects on physicians, their families and patients, and the health care system, with a substantial negative impact on the health economy.^5,6^ The COVID-19 pandemic exacerbated burnout and stress, with increased depression, insomnia, anxiety, and distress observed in physicians.^7,8^ Thus, interventions are needed to decrease psychological symptoms, improve sleep, and prevent burnout to avert major detrimental outcomes, such as adverse impacts on care quality, patient experience, and care access, in our health care system.^6,9^

Previous studies^10,11,12,13^ have reported that lack of organizational measures is a major factor in physician burnout. However, individual approaches that are aligned with system-level measures are also needed.^13,14,15^ Thus, specific programs are needed for physicians to increase their wellness. Here, we focus on the mental well-being of physicians, the dimension most frequently used to operationalize physician wellness.^16^ This includes measures of burnout, depression, stress, or distress, and work attitudes, such as work or job satisfaction, career satisfaction, and organizational or career commitment. Sleep quality and optimism are included as related aspects.^16^ Several wellness approaches have been previously introduced for physicians, including mindfulness practices, meditation, stress management programs, and small group discussions.^4,10,13,14^ Most of these studies were correlational and evaluated a limited number of individual parameters; some of these studies reported small but significant effects.^17^ Overall, the previous studies indicate that individual interventions are potentially feasible and can, under the right conditions, result in favorable outcomes for physician wellness.

In a 2022 survey-based study^15^ of a group of health care professionals primarily consisting of physicians, we reported significant positive outcomes following an online version of a comprehensive wellness program called Sudarshan Kriya Yoga (SKY), which was consistent with a pilot study.^30^ SKY includes gentle yoga stretches, breathing techniques that have a unique rhythmic breathing and meditation exercise, and cognitive coping and stressor evaluation strategies.^17^ No previous knowledge or experience with similar programs is necessary to participate in SKY, and the program can be conducted online in its entirety. Previous studies have shown that SKY decreased posttraumatic stress among war veterans,^18^ relieved stress, anxiety, and depression in prisoners,^19^ and significantly decreased clinical depression.^20,21^ In addition, SKY increased various wellness parameters in university students more effectively than 2 other wellness programs.^22,23^ There is also emerging knowledge regarding the potential mechanisms of SKY, such as reduced biomarkers of stress (eg, cortisol),^24,25^ improved respiratory function,^18^ increased heart rate variability,^26^ as well as epigenetic effects in circulating immune cells.^27^ Here, we present the results of a randomized clinical trial that aimed to assess the potential efficacy of SKY to improve wellness and mitigate burnout in physicians.

## Methods

### Study Design

This randomized clinical trial was conducted online between November 2021 to March 2022 with participants from all over Turkey (36 provinces), Germany (1 province), and Dubai (1 province). The trial protocol and statistical analysis plan was approved by the Izmir Biomedicine and Genome Center institutional review board (Supplement 1). All participants provided written informed consent. Two researchers (AK and DS) were responsible for enrolling and randomizing the participants to the 2 groups. This study followed the Consolidated Standards of Reporting Trials (CONSORT) reporting guideline.^28^ The study compared SKY with the control group after a 3-day intervention and evaluated measures at baseline, after training (posttraining), and in the primary end point at 8 weeks (postintervention).

### Participants

Practicing physicians from 36 provinces across Turkey, as well as 1 each from Germany and Dubai, were recruited to the study. Participants were included if they were a practicing physician, aged 25 to 65 years, had the ability to give informed consent, were interested in being part of a study to evaluate breath or meditation-derived exercises, and were willing to do a relaxation exercise every day for 2 months. Individuals were excluded if they had psychiatric or major somatic illnesses (eg, schizophrenia or schizoaffective disorder, bipolar disorder, posttraumatic stress disorder, uncontrolled hypertension, lung disease, liver disease, cancer, or heart disease) or were currently maintaining a regular mind-body program practice (eg, meditation, yoga, or breathing techniques).

### Recruitment and Randomization

**Figure 1.**
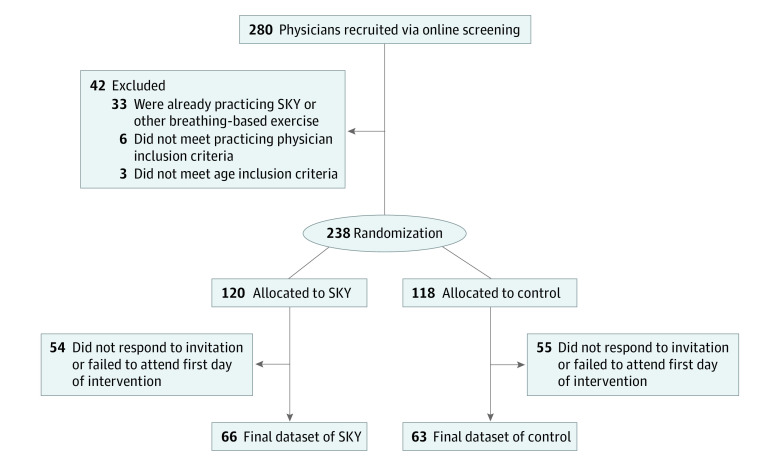
Diagram Summarizing the Flow for Recruitment, Allocation, and Final Data Collection SKY indicates Sudarshan Kriya Yoga.

A previous study^23^ comparing SKY with an active control group displayed postintervention effect sizes ranging 0.3 to 0.5 for our core outcomes (ie, perceived stress, depression, and sleep disturbance). Therefore, we set our target sample size to 100 individuals per group based on an estimated effect size of 0.4 for differences between the study groups, power of 0.8, and an α of .05, with the primary outcome of stress, depression, and insomnia. Upon announcements on relevant websites and social media, 280 physicians initially expressed interest in the study and provided informed consent as part of the screening surveys. Figure 1 illustrates how the final participant sample was compiled. From the initial pool of interested participants, 42 of them did not fulfill the inclusion or exclusion criteria. Participants were then randomly assigned 1:1 to the control group using stress management education (SME) or to the SKY group via a computer algorithm. Eligible participants were invited to enroll in the 3-day interventions and commit to the tests and attendance schedule in the follow-up sessions for 8 weeks. Of 238 participants, 133 further accepted the invitation and 129 joined the study. Of the remaining 109 individuals, 105 did not respond to the invitation to participate in the training groups and 4 failed to attend the first day of training. All participants who started the program completed the study filling standardized scales online at baseline, posttraining, and postintervention. The postintervention test included questions about home practice frequency for dose-response analysis.

### Interventions

The SKY intervention was 1.5 hours online for 3 consecutive days in the evenings delivered by 2 instructors. This is a shorter form of the traditional SKY program that was taught face-to-face prior to the pandemic.^20,21,22,23,24,25,26,27^ It was also shorter than the pilot online study conducted during the early part of the pandemic,^30^ based on the feedback on time pressure received during a pilot program in Europe.^15^ The sessions included gentle stretches (eg, office yoga in a sitting position), specific breathing exercises and meditation, and discussion of cognitive or behavioral coping skills (a compact version of the program that was previously described).^17^ Briefly, after the stretches, the participants practiced the following exercises: (1) 3-stage pranayama with ujjayi or victory breath, (2) 3 sets of Bhastrika or bellow’s breath, and (3) Sudarshan Kriya. The exercises were done in a sitting position that was comfortable, either in a chair or on the floor, and the eyes were kept closed throughout the practice. After the 3-day program, the participants were asked to implement a daily home practice (approximately 30 minutes per day); in addition, there were weekly online follow-up sessions (group practice and discussion) that lasted for about 1 hour during the 8 weeks of follow-up.

Similar to the SKY group, participants in the control group had online sessions with 2 instructors for 1.5 hours a day on 3 consecutive evenings. The training consisted of video education resources that explained how one can reduce psychological distress, including cognitive coping strategies, in a group discussion format. After the 3-day intervention, participants were asked to implement these points in their daily life; furthermore, they met online once a week with 2 instructors in 1-hour follow-up sessions (group discussion and sharing) for 8 weeks.

### Measures

The primary outcomes of this study were stress and depression measured by the 42-item Depression, Anxiety, and Stress Scale (DASS-42); and insomnia measured by the Regensburg Insomnia Scale (RIS) with primary end point at postintervention (8 weeks). Additionally, related outcomes were also included in the primary analyses: anxiety (DASS-42); optimism (Life Orientation Test—Revised [LOT-R]); professional fulfillment, work exhaustion, interpersonal disengagement, and overall burnout (Professional Fulfillment Index [PFI]); and self-reported professional errors (Self-Reported Professional Error Questionnaire).

#### DASS-42

The survey consists of 42 items (14 items for each scale), a set of 3 self-report scales designed to measure negative emotional states of depression, anxiety, and stress.^29^ On a scale of 0 to 3 for intensity and frequency (0, never; 3, most of the time), participants rate how much they have experienced each symptom during the past week. A sum score from the 14 items for each scale was calculated (range, 0 to 42), where a higher score indicated more severe depression, anxiety, or stress symptoms.

#### LOT-R

The LOT-R is a 10-question self-report scale that measures the level of optimism and expectations about generally positive outcomes.^31^ Participants were asked to answer the questions on a 0 to 4-point scale (0, strongly disagree; 4, strongly agree). The sum score from 6 specific items is calculated to obtain the overall score; 4 are filler items.

#### Professional Fulfillment Index (PFI)

The survey consists of 16 items that report on professional fulfillment, work exhaustion, interpersonal disengagement, and overall burnout.^32^ Answers to the items are arranged on scales of 0 to 4 or 0 to 5, depending on the subscale. Final scores are obtained by finding the mean of the scores within each subscale. The burnout score is obtained by fusing work exhaustion and depersonalization subscales.

#### Self-Reported Professional Error Questionnaire

This short survey aims to measure self-reported errors in diagnosis, medication orders, and tests; it is a 4-item questionnaire originally developed to accompany the PFI.^32^ Responses relate to most recent occurrences (from last week to lifetime) on a 6-point scale. Sum score from the 4 items is calculated to obtain the overall score.

#### Regensburg Insomnia Scale (RIS)

The survey consists of 10 items and is a self-rating scale to measure cognitive, emotional, and behavioral aspects of psychophysiological insomnia.^33^ On a scale of 0 to 4 for intensity and frequency, participants rate how much they have experienced each symptom for the last 4 weeks. The sum score from the 10 items is calculated to obtain the overall score.

### Statistical Analyses

Descriptive data of the outcome measures across groups and the 3 time points were provided as mean (SD) and median (IQR), and we tested for differences between groups using independent sample *t* test and Wilcoxon rank sum test, depending on the data. We presented the number of females stratified by group, with percentages, and compared the sex-distribution across the 2 groups using Fisher exact test. We calculated a standardized mean difference score for each outcome measure (Cohen *d*) at posttest and follow-up. Cohen *d* values ranging from 0.01 to 0.20, 0.20 to 0.50, 0.50 to 0.80, and more than 0.80 correspond to very small, small, moderate, and large effect size, respectively. Due to lack of baseline data from the 105 individuals that were randomized but did not participate in the intervention, including the baseline assessments, we conducted per protocol analyses instead of intention to treat (ITT) analyses.

In the primary analyses, we used mixed linear regression to examine whether changes in the outcome measures were the result of the interaction between group and time. We included group (SKY or control), time (baseline, posttraining, and postintervention), and an interaction term between group and time (group × time) as fixed effects, and participant’s identification as random effect. We tested all models for normal distribution of the residuals and homoscedasticity. Results are presented as marginal mean differences at posttraining and postintervention, with 95% CIs and *P* values for the time by group interaction. We illustrated the mean (95% CI) of the outcome measures across the 3 time points using Prism version 9 (GraphPad).

In the secondary exploratory and subgroup analyses, we further examined the possible impact of the SKY practice frequency by restricting the SKY group to those who performed the practice 3 or more times per week (high adherence group) and using the similar mixed linear regression model approach (eTable 1 in Supplement 2). Additionally, we categorized the SKY group into those who performed the practice 1 to 2 times per week (low adherence group) and compared them with the high adherence group and assessed the interaction between this categorization and time in a mixed linear model. Adherence group (1 to 2 times per week and 3 or more times per week), time (baseline, posttraining, and postintervention), and an interaction term between adherence group and time (group × time) were included as fixed effects, and participant’s identification was included as random effect. The control group was not included in these latter analyses because no measures for the frequency of performing the program were available from that group (eTable 2 and eFigures 1 to 3 in Supplement 2). These analyses are exploratory in nature, and as such, we have not applied multiple comparison corrections to the *P* values.

Because there were few male participants in the study, we were not able to test the interaction with sex. Furthermore, because of the observed significant difference in the proportion of males between the 2 groups, we performed sensitivity analyses where we reran the main analyses adjusting for sex (eTable 3 in Supplement 2). We performed all analyses using Stata SE version 17.0 (StataCorp), and statistical significance was set to *P* < .05. All tests were 2-tailed, and the statistical analyses were conducted from March to November 2023

## Results

### Demographic Information

Of 129 participants, 115 (89.2%) were females and 14 (10.8%) were males. All participants (129 [100%]) were Turkish, from 36 provinces across Turkey, and 1 each from Germany and Dubai. Approximately 50% of participants were from the 3 largest cities in Turkey (Istanbul, Ankara, and Izmir). Two participants (1.6%) were aged younger than 30 years, 34 participants (26.4%) were aged 30 to 39 years, 44 participants (34.1%) were aged 40 to 49 years, 40 participants (31.0%) were aged 50 to 59 years, and 9 (7.0%) were aged 60 to 65 years. Additionally, 95 participants (73.6%) were married, 31 (24.0%) were single, and 3 (2.3%) elected not to declare any marital status. The participants’ specialties included family medicine (25 [19.4%]), general practitioner (12 [9.3%]), gynecology and obstetrics (11 [8.5%]), anesthesiology (10 [7.8%]), internal medicine (9 [7.0%]), pediatrics (8 [6.2%]), radiology (8 [6.2%]), pulmonology (7 [5.4%]), physiotherapy and rehabilitation (6 [4.7%]), infectious diseases (4 [3.1%]), emergency medicine (3 [2.3%]), medical aesthetics (3 [2.3%]), ophthalmology (3 [2.3%]); dermatology (2 [1.6%]), hemodialysis specialist (2 [1.6%]), medical oncology (2 [1.6%]), nuclear medicine (2 [1.6%]), radiation oncology (2 [1.6%]), neurology (2 [1.6%]), occupational physician (2 [1.6%]), cardiovascular surgery (1 [0.8%]), gynecologic oncology (1 [0.8%]), microbiology (1 [0.8%]), pediatric surgery (1 [0.8%]), thoracic surgery (1 [0.8%]), and otorhinolaryngologist (1 [0.8%]). Sixty-three participants (48.9%) were included in the SME control group and 66 (51.1%) were included in the SKY group.

### DASS-42

**Figure 2.**
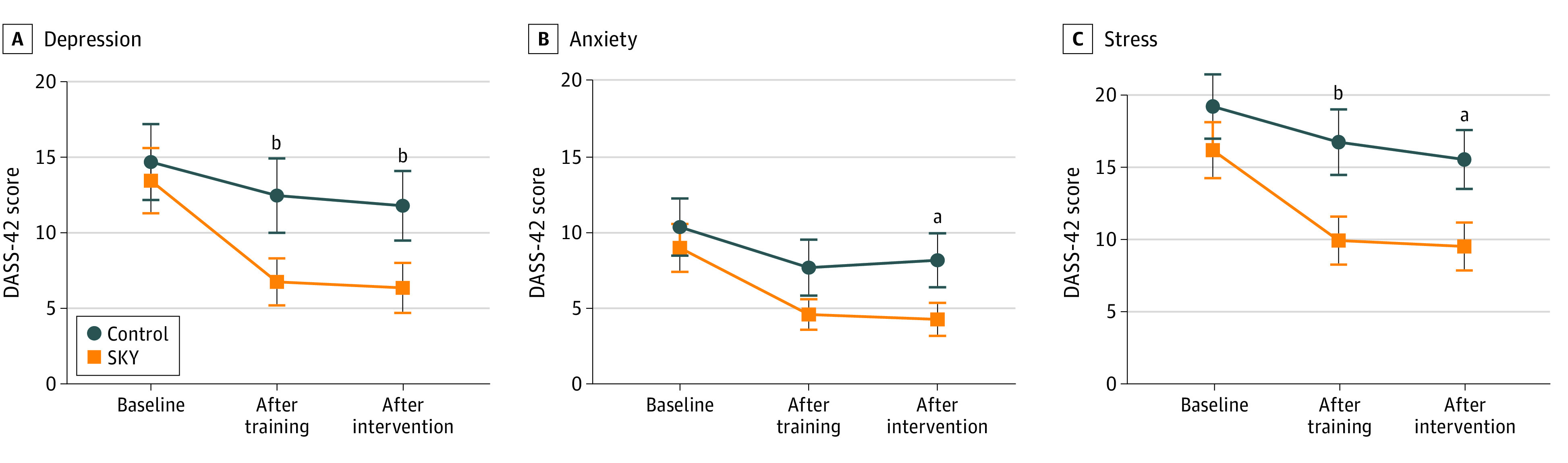
Depression Anxiety and Stress Scale (DASS-42) Outcomes by Group and Time Points Points indicate means and whiskers and 95% CI. ^a^*P* < .05. ^b^*P* < .01.

Compared with the SME control group, participants in the SKY group had significantly decreased stress on the DASS-42 at posttraining (difference, −6.8 points; 95% CI, −9.6 to −4.1 points; *P* = .006) and at postintervention (difference, −6.0 points; 95% CI, −8.8 to −3.3 points; *P* = .03) and significantly decreased depression at posttraining (difference, −5.7 points; 95% CI, −8.6 to −2.8 points; *P* < .001) and postintervention (difference, −5.4 points; 95% CI, −8.3 to −2.5 points; *P* < .001). SKY significantly decreased anxiety at postintervention but not at posttraining (Table and Figure 2). The marginal means difference of all the outcome measures were significantly higher in the control group compared with SKY at both postintervention and follow-up, with values ranging from 3.1 points (95% CI, −5.2 to −0.9 points) for anxiety score at postintervention to 6.8 points (95% CI, −9.6 to −4.1 points) for stress score at posttest. Moderate to large effect sizes were suggested by Cohen *d* values. The results from the additional analyses pertaining to the practice frequency, between high and low adherence groups, suggested no significant differences (eTable 2 and eFigure 1 in Supplement 2).

**Table.** Comparisons Between Groups in Characteristics and Outcome Measures Across the Study Period, and Results From Mixed Linear Models With Group by Time Interactions to Examine Intervention Effects

Characteristics and outcome measures	Score, mean (SD)		Cohen d	Marginal mean difference (95% CI)^a^	***P* value for group × time** ^b^
	SKY	Control			
Participants, No. (%)	66 (51.1)	63 (48.9)	NA	NA	NA
Sex, No. (%)					
Female	54 (81.8)	61 (96.8)^c^	NA	NA	NA
Male	12 (18.2)	2 (3.2)	NA	NA	NA
**DASS-42**					
Depression					
Baseline	13.4 (8.8)	14.7 (9.9)	NA	NA	NA
Posttraining	6.8 (6.3)	12.5(9.8)^d^	−0.70	−5.7 (−8.6 to −2.8)	.001
Postintervention	6.4 (6.7)	11.8 (9.1)^d^	−0.68	−5.4 (−8.3 to −2.5)	.001
Anxiety					
Baseline	8.9 (6.4)	10.3 (7.5)	NA	NA	NA
Posttraining	4.5 (4.1)	7.6 (7.3)^c^	−0.52	−3.1 (−5.2 to −0.9)	.12
Postintervention	4.2 (4.4)	8.1 (7.1)^d^	−0.66	−3.9 (−6.0 to −1.7)	.02
Stress					
Baseline	16.2 (7.9)	19.2 (8.9)^e^	NA	NA	NA
Posttraining	9.9 (6.8)	16.7 (9.0)^d^	−0.86	−6.8 (−9.6 to −4.1)	.006
Postintervention	9.5 (6.8)	15.5 (8.1)^d^	−0.81	−6.0 (−8.8 to −3.3)	.03
**PFI**					
Professional fulfillment					
Baseline	1.83 (0.82)	1.90 (0.97)	NA	NA	NA
Posttraining	2.24 (0.87)	1.96 (0.91)	0.31	0.28 (−0.03 to 0.59)	.005
Postintervention	2.29 (0.83)	1.98 (1.03)	0.33	0.31 (0 to 0.62)	.002
Work exhaustion					
Baseline	2.17 (0.98)	2.19 (1.01)	NA	NA	NA
Posttraining	1.51 (0.91)	1.97 (1.09)^e^	−0.45	−0.45 (−0.80 to −0.11)	.008
Postintervention	1.46 (0.88)	1.82 (1.14)^e^	−0.35	−0.36 (−0.70 to −0.01)	.04
Interpersonal disengagement					
Baseline	1.42 (0.80)	1.50 (1.00)	NA	NA	NA
Posttraining	1.00 (0.70)	1.41 (0.90)^c^	−0.51	−0.41 (−0.71 to −0.11)	.03
Postintervention	0.89 (0.77)	1.34 (0.99)^c^	−0.50	−0.45 (−0.75 to −0.15)	.02
Burnout					
Baseline	1.72 (0.80)	1.78 (0.91)	NA	NA	NA
Posttraining	1.21 (0.69)	1.63 (0.91)	−0.53	−0.43 (−0.72 to −0.14)	.007
Postintervention	1.12 (0.73)	1.53 (0.98)	−0.48	−0.41 (−0.70 to −0.12)	.01
**Medical errors, median (IQR** **)**					
Baseline	2 (0-3)	2 (0-4)	NA	NA	NA
Posttraining	2 (0-3)	2 (0-4)	NA	−1.00(−1.96 to −0.05)	.17
Postintervention	1 (0-3)	2 (0-4)	NA	−0.99 (−1.95 to −0.04)	.18
**LOT-R**					
Baseline	13 (2.9)	13.4 (4.2)	NA	NA	NA
Posttraining	14.2 (3.8)	13.8 (4.0)	0.10	0.4 (−0.9 to 1.7)	.20
Postintervention	14.8 (3.4)	14.1 (4.1)	0.18	0.7 (−0.6 to 1.9)	.09
**RIS**					
Baseline	14.8 (5.6)	13.1 (6.4)	NA	NA	NA
Posttraining	12.8 (5.6)	12.2 (5.9)	0.09	0.5 (−1.4 to 2.5)	.14
Postintervention	11.8 (4.9)	12.1 (6.5)	−0.05	−0.3 (−2.3 to 1.7)	.01

Abbreviations: DASS, Depression Anxiety and Stress Scale; LOT-R, Revised Life Orientation Test; NA, not applicable; PFI, Professional Fulfillment Index; RIS, Regensburg Insomnia Scale; SKY, Sudarshan Kriya Yoga.

^a^ Marginal mean difference from mixed linear regression analyses (SKY vs control group).

^b^ *P* value from interaction term (group × time) from mixed linear regression analyses.

^c^ *P* < .01 from *t* tests for normally distributed data and Wilcoxon rank sum test for medical error.

^d^ *P* < .001 from *t* tests for normally distributed data and Wilcoxon rank sum test for medical error.

^e^ *P* < .05 from *t* tests for normally distributed data and Wilcoxon rank sum test for medical error.

### LOT-R

**Figure 3.**
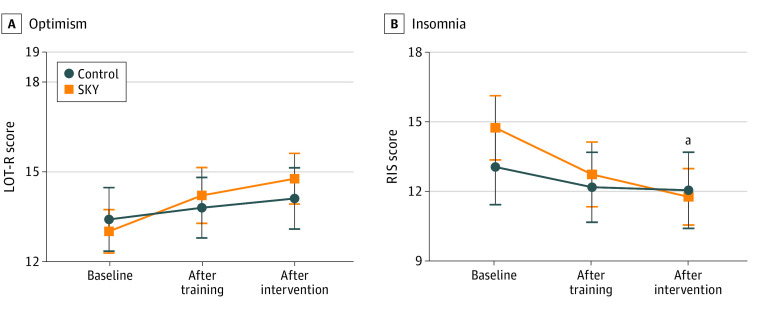
Revised Life Orientation Test (LOT-R) and Regensburg Insomnia Scale (RIS) Outcomes by Group and Time Points Points indicate means and whiskers indicate 95% CI. ^a^*P* < .05.

The data suggested no intervention effect on level of optimism and no differences between the SKY and control group at any of the 3 time points (Table and Figure 3). The high adherence group had a significant intervention effect at postintervention compared with the control group (eTable 1 in Supplement 2). Furthermore, there was a significant adherence group by time interaction (eTable 2 and eFigure 2 in Supplement 2). The marginal mean difference on the LOT-R scale in the control group compared with the high adherence group in SKY was −1.5 points (95% CI, −3.1 to 0.2 points).

### RIS

The group by time interaction suggested no intervention effect on the insomnia scale from baseline to posttraining, but a significant effect from baseline to postintervention (difference, −0.3 points; 95% CI, −2.3 to 1.7 points; *P* = .01) (Table and Figure 3). There was a larger decrease in symptoms in the SKY group from baseline to postintervention. No marginal mean differences were observed between the 2 groups at posttest and follow-up, and Cohen *d* suggested very small effect sizes. The results from the subgroup analyses suggested no differences between the low and high adherence groups (eTables 1 and 2 and eFigure 2 in Supplement 2).

### PFI and Self-Reported Medical Errors

**Figure 4.**
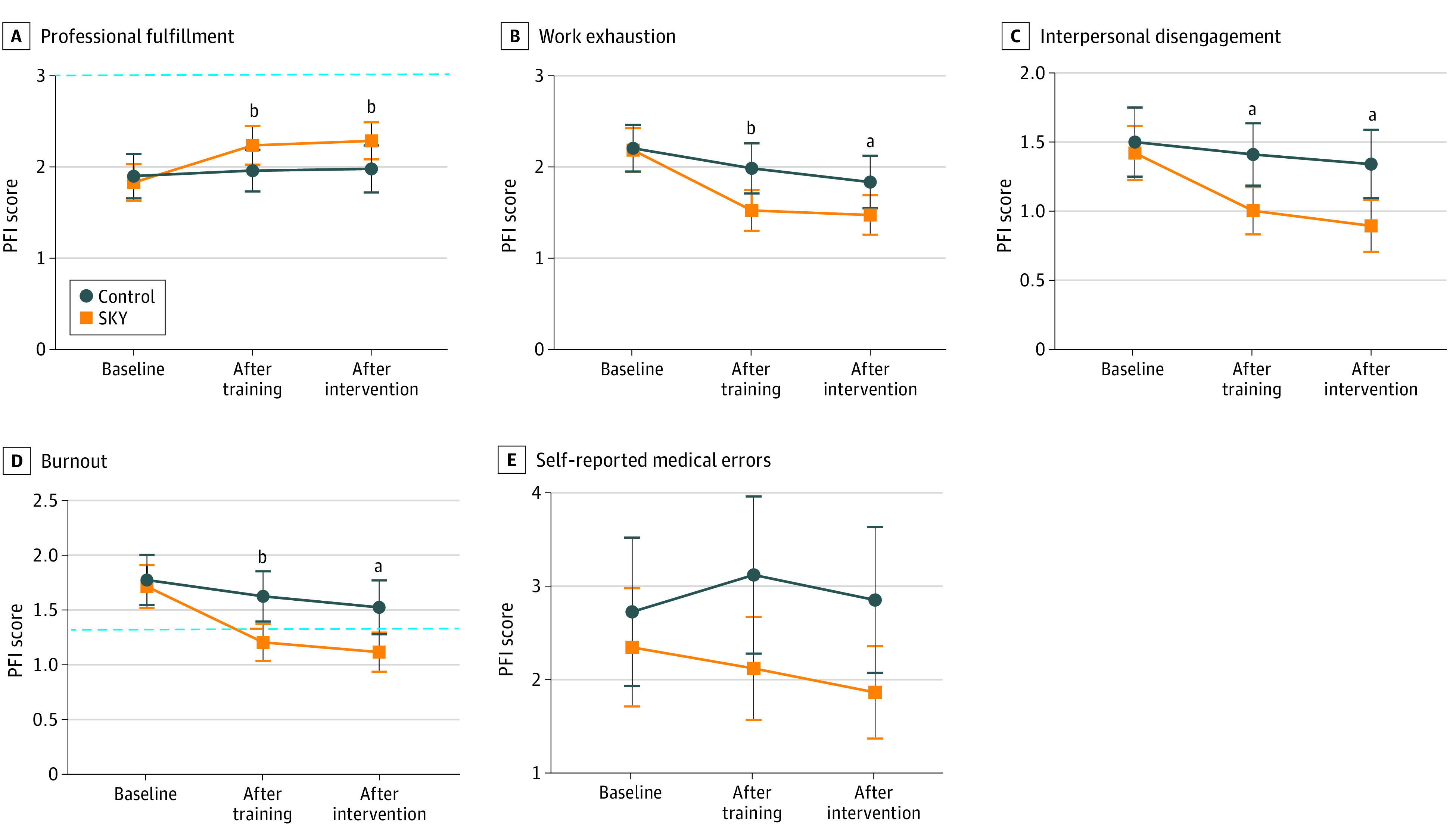
Professional Fulfillment Index (PFI) Outcomes by Group and Time Points For the burnout and professional fulfillment subscales, cutoff values of 1.33 and 3.00, respectively, are indicated in the corresponding graph with the dashed blue horizontal line. Points indicate means and whiskers indicate 95% CIs. ^a^*P* < .05. ^b^*P* < .01.

The results on the PFI outcomes suggested a significant group by time interaction on professional fulfillment, interpersonal disengagement, work exhaustion, and burnout from baseline to posttest and from baseline to follow-up (Table and Figure 4). We observed no intervention effect on self-reported medical errors. The control group demonstrated higher work exhaustion, interpersonal disengagement, burnout and self-reported medical errors at posttest and follow-up, and borderline lower professional fulfilment at follow-up (marginal mean difference, −1.9 points; 95% CI, −3.7 to 0 points). Cohen *d* suggested small to moderate effect sizes. The burnout and professional fulfillment scores are typically presented as dichotomous categories.^33^ For the burnout scale, at baseline both groups were significantly above the cutoff value of 1.33 points; whereas the scores for the SKY group decreased to below this value at follow-up and remained lower at postintervention, the control group displayed higher values than the cutoff through the study (Table and Figure 4). For the professional fulfillment subscale, the values were below the cutoff of more than 3 points for all time points for both groups (Table and Figure 4).

The adherence group by time interaction suggested a larger follow-up effect size of the intervention on interpersonal disengagement, burnout, and self-reported medical errors in the high vs low adherence group (eTable 2 and eFigure 3 in Supplement 2). Restricting the linear mixed model to the high adherence group demonstrated a significant intervention effect size on self-reported medical errors, where the control group reported 1.4 points (95% CI, 0.1 to 2.6 points) more self-reported medical errors per week, compared with the high adherence group (eTable 1 in Supplement 2). Sensitivity analyses with additional adjustments for sex did not affect the results on any of the outcome measures (eTable 3 in Supplement 2).

## Discussion

In this randomized clinical trial of physicians, participants in the SKY group displayed significantly reduced rates of depression, anxiety, and stress compared with control participants. Additionally, there were significant increases in professional fulfillment as well as decreases in work exhaustion, interpersonal disengagement, and burnout in the SKY group but not the control group. Furthermore, there was a significant improvement in insomnia symptoms throughout the 8-week follow-up. These findings suggest that SKY could be coupled with institutional measures for optimum mitigation of physician burnout.

Consistent with other reports on gender differences on the frequency of wellness practice use,^34^ our study had a higher number of female participants, which could be because distress and burnout has been found to be significantly higher in female workers compared with their male counterparts.^35,36^ This could lead to increased interest in seeking interventions designed to mitigate these symptoms. Our data suggest that SKY is effective in decreasing psychological distress and burnout on multiple parameters and may be a potential tool to mitigate burnout for female physicians. Future studies with higher numbers of male participants are required to reliably test the specific efficacy of the intervention among male physicians.

Our analysis indicated that the frequency of SKY practice may determine the outcome for some of the measures; however, SKY still appeared to be significantly beneficial even when home practices was missed a few days a week. Given the small numbers of participants in the supplementary analyses, the findings should be interpreted with caution. Nevertheless, it is interesting to note that while in the whole SKY group the differences compared with the controls did not reach significance for LOT-R and self-reported medical errors, there were significant differences when comparing the high adherence group to controls (Supplement 2). In contrast, for measures that were strongly affected by SKY in the whole group, such as the DASS-42 scale, there were no further improvements by frequency of practice. Altogether, these data suggest that a more regular practice regimen is warranted for optimal benefit from the SKY program.

We noted that a substantial number of participants who showed interest in the study and were randomized, did not participate at the end, which led to lack of available data concerning the baseline questionnaire. The individuals who initially showed interest participating struggled to make the time commitment when the schedules were announced. Therefore, we were unable to run ITT analyses using baseline data from these individuals. The preintervention attrition of participants also contributed to a lower-than-anticipated number of participants, and we did not meet our initial recruitment goal. This could have increased the likelihood of committing a type 2 error. Nevertheless, once enrolled in the study and participated in the first session, all participants finished the study.

Our study has several strengths. First, to our knowledge, this study is one of the largest randomized clinical trials to assess the efficacy of a program that aims to increase physician wellness. Second, there was an active control group that interacted at similar time periods with instructors, in a manner similar to the SKY group. Third, we used internationally validated scales, of which some have been specifically developed to monitor physician wellness and performance. Fourth, there was no attrition during the 8-week follow-up, attesting to the high acceptability of the program.

### Limitations

This study has limitations. First, as noted above, the participant group was largely female, and it would be beneficial to increase the percentage of male participants in future studies. Second, it is possible that the participants were self-selected among a group that was more motivated than an average cross-section of physicians and benefited more from the intervention. Third, we did not have the resources to examine objective measures, such as daily measures of saliva cortisol, inflammatory cytokines, or heart rate variability, to determine if they are associated with our findings from the various scales in this study. Fourth, due to limited numbers of participants, we could not explore in detail the dose response effects of the SKY intervention. Fifth, it would have been ideal to compare SKY with an active control group who had a home practice of equal length.

## Conclusions

In this randomized clinical trial, we found that SKY was a practical program that was safe, easily implemented, and highly accepted. SKY significantly reduced psychological distress and burnout and increased wellness in physicians on several parameters. Given the high personal and financial toll of physician burnout worldwide, these findings suggest that SKY could be considered as a preventive measure or used to mitigate stress and burnout in physicians. Given its practicality, SKY may be a feasible tool to mitigate stress and burnout in other vulnerable professions.

## Notes

Supplement 1.
Trial Protocol

Supplement 2. **eTable 1.** Mixed Linear Regression Model and High Adherence SKY Group and the Control Group **eTable 2.** Mixed Linear Regression Model and High and Low Adherence SKY Groups **eTable 3.** Sensitivity Analyses **eFigure 1.** DASS-42 Outcomes by Adherence Group and Time Points **eFigure 2.** LOT-R and RIS Outcomes by Adherence Group and Time Points **eFigure 3.** PFI Outcomes by Adherence Group and Time Points

Supplement 3.
Data Sharing Statement
