# Supplementary material for: Sudarshan Kriya Yoga Breathing and a Meditation Program for Burnout Among Physicians: A Randomized Clinical Trial
Source: JAMA Netw Open. 2024 Jan 31;7(1):e2353978. doi: 10.1001/jamanetworkopen.2023.53978 (PMC10831575; doi:10.1001/jamanetworkopen.2023.53978)
Supplement: Supplement 1. — Trial Protocol [file jamanetwopen-e2353978-s001.pdf]

IZMIR BIOMEDICINE and GENOME CENTER
NON-INTERVENTIONAL RESEARCH ETHICS COMMITTEE
APPLICATION FORM

Protocol Number : 2021-015 Application Date : 23/05/2021

A. FULL TITLE OF THE PROTOCOL

A Comprehensive Meditation and Breath Program (SKY) as an Adjunct Tool to Increase Resilience and Wellness in Frontline Healthcare Workers – A Randomized Pilot Study

Nature of the Study: Individual Research Project

B. RESEARCH TEAM

Principal Investigator

Name: Duygu SAĞ	Title : Assist. Prof. Dr.
Institution: İzmir Biomedicine and Genome Center	Area of Expertise: Immunology
Phone : +90 0532 6387999	e-mail: duygu.sag@ibg.edu.tr

Team Members

Name	Title	Institution / Area of Expertise	E-mail/Phone	Title in the project
Fahri Saatcioglu	Prof.	University of Oslo/ Norway /Cancer Biology / Breath and meditation (SKY) trainer	fahri.saatcioglu@ibv.uio.no	Associate investigator
Burcu Cirit	Dr.	Health Sciences University, Ataturk Thoracic Diseases Education and Research Hospital, Ankara / Thoracic diseases / SKY trainer	drcirit@hotmail.com	Associate investigator
Gayem Köprücü Süzer		American Hospital / Koç University Hospital, Istanbul / Speech and Language Pathology / SKY trainer	gayemkoprucu@gmail.com	Associate investigator

C. INFORMATION ABOUT RESEARCH

Research Center: Online

Type / Subject / Design of Research: Survey studies

Rationale / Purpose of the Research:

The current COVID-19 pandemic has resulted in great physical and mental strain on frontline healthcare professionals who are already at high risk for these conditions, including significantly increased anxiety and depression. This is due to many factors, including lack of adequate protective equipment, fear of contracting the virus, fear of spreading it to loved ones, and the necessity to frequently make important decisions about the life and death of patients. Therefore, there is an urgent need to intervene to reduce the impact of these factors on the psychological health of health workers to protect them and the health system from harmful consequences. Various tools to regulate and support psychological health have been developed for pandemics, but their effectiveness has not yet been conclusively tested. Among the most widely used methods in this context are meditation and breathing programs, one of which is Sudarshan Kriya Yoga (SKY). SKY is a comprehensive program that uses specific breathing exercises to eliminate stress, support various organs and systems in the body, transform disturbing emotions and promote inner peace. Research on SKY, published in international scientific journals, has shown significant positive effects on various aspects of participants' physiology and psychology. The aim of this project is to systematically examine the possible impact of SKY on the psychological health of healthcare professionals working with the COVID-19 pandemic. The studies will include psychological assessment through self-report standardized questionnaires on various aspects of health and resilience. Our hypothesis is that SKY will reduce psychological distress, increase resilience, and trigger positive changes.

Our preliminary data from surveys conducted after the SKY programs offered to over 1700 frontline health professionals in Turkey and Portugal during the pandemic supports our hypothesis that SKY can make significant tangible contributions to various aspects of wellness in frontline healthcare workers. This planned pilot clinical trial will test this hypothesis and the feasibility of SKY in this context; if the results are positive, this will be the basis for larger clinical trials.

Materials and Methods:

Volunteers who are medical doctors working with the COVID-19 pandemic will be divided into 2 groups: experimental group and control group. EXPERIMENTAL GROUP: The SKY program will be implemented. It includes gentle stretches, specific breathing exercises, and cognitive coping and stressor assessment strategies. Subjects are first trained in SKY for 3 consecutive days for 1.5 hours each. They then apply what they have learned in during the intervention as a daily home practice (about 30 minutes) throughout the research period. In addition, subjects participate in weekly group follow-up sessions of approximately one hour that includes group practice and a Q&A session regarding their home practice is.

Breathing techniques that are part of SKYare: (a) Ujjayi or "Victory Breath" with ’three-stage-pranayama practice, (b) three sets of Bhastrika or "Bellows Breath", and (c) SK, a rhythmic breath meditation program, practiced in this order. All practices are done in a sitting position on a chair or on the floor.Eyes are kept closed throughout the sessions.

CONTROL GROUP: Stress management training (SMT) will be is conducted for 1.5 hours a day for three consecutive days. The training will consist of various video resources explaining how one can reduce stress, including cognitive coping strategies (e.g. https://www.youtube.com/watch?v=hpmST69N3Zk). Similar to the experimental group, there will be weekly follow-up sessions for the control group, lasting approximately one hour, where the material

used during the main 3-day program will be reviewed and questions answered.

A total of 200 health professionals will be randomized into two study arms of 100 each: experimental group and control group. The target for each group is 80 subjects, but to account for dropout/exclusion, 100 subjects per group will be included (with an expected dropout/exclusion rate of 20%). Both groups will be pretested at the time of recruitment into the study. The experimental group and the control group will be divided into two and the trainings will be delivered in two groups.

Two days after the training, the tests will be done again (Posttest 1) in a similar way to the pretest. Eight weeks after Posttest 1, Posttest 2 will be performed using the same parameters. The data obtained will be compared within and between the experimental and control groups at these three different times. Below is a brief flowchart of the study timeline:

Weeks 1-3 Inclusion of participants
Week 4 PreTest
Week 4/5 SKY
Week 4/5 Posttest 1
Week 12/13.
Week Posttest 2

Psychological well-being parameters will be assessed using standardized questionnaires at the time points described above. The questionnaires to be used are described in the "Procedures and Variables to be used in the Study" section.

**Estimated Number of Volunteers:**	200
**Volunteer Group Characteristics:**	- >Over 18 years of age<br>- Female and Male<br>- Healthy<br>- Active physicians and nurses<br>- (100 subjects in each of the control and the experimental group)

Inclusion and Exclusion Criteria:

Inclusion criteria: 25-65 of age; ability to speak/read Turkish and give informed consent; interest in being part of a study to evaluate breath-derived exercises, willingness to do some form of relaxation exercise every day for 2 months, active practicing physician.

Exclusion criteria: Psychiatric illness or other major illness such as bipolar disorder, chronic PTSD, schizophrenia or schizoaffective disorder, uncontrolled hypertension, lung disease, liver disease, cancer, or heart disease. Currently practicing a mind-body program regularly, such as meditation, yoga and breathing techniques. Note: These criteria will be specified in a form that the subjects will fill in when they apply to be included in the study.

Information obtained during the application will be stored in a keyword-protected file on a keyword-protected computer until the end of the study and then deleted.

Control Group : Inclusion and exclusion criteria for the control group is identical with the experimental group.

Procedures and Variables to be used in the Research:

The parameters of psychological well-being in the subjects will be assessed by administering the standardized questionnaires listed below:

1. Depression, Anxiety and Stress Scale (DASS)-42: A set of three self-report scales designed to measure the negative emotional states of depression, anxiety and stress (Lovibond and Lovibond, 1995).

2. Professional Satisfaction Index (PFI): A brief instrument to assess both burnout and professional satisfaction in physicians (Trockel et al., 2018).

3. Life Orientation Test - Updated (LOT-R): A 10-item self-report scale measuring expectations about positive outcomes in general (Scheier et al., 1994).

4. Regensburg Insomnia Scale (RIS): A self-report scale to assess cognitive, emotional, and behavioral aspects of psychofisological insomnia (Crönlein et al., 2013)

Estimated Research start date: 15/06/2021

Estimated duration of the research: 6 Months

References:

1- Crönlein, T., Langguth, B., Popp, R., Lukesch, H., Pieh, C., Hajak, G., and Geisler, P. (2013).

2- Regensburg Insomnia Scale (RIS): a new short rating scale for the assessment of psychological symptoms and sleep in insomnia; study design: development and validation of a new short self- rating scale in a sample of 218 patients suffering from insomnia and 94 healthy controls. Health Qual Life Outcomes 11, 65.

3- Doria, S., de Vuono, A., Sanlorenzo, R., Irtelli, F., and Mencacci, C. (2015). Anti-anxiety efficacy of Sudarshan Kriya Yoga in general anxiety disorder: A multicomponent, yoga based, breath intervention program for patients suffering from generalized anxiety disorder with or without comorbidities. J Affect Disord 184, 310-317.

4- Dutheil, F., Mondillon, L., and Navel, V. (2020). PTSD as the second tsunami of the SARS-Cov-2 pandemic. Psychol Med, 1-2.

5- Goldstein, M.R., Lewin, R.K., and Allen, J.J.B. (2020). Improvements in well- being and cardiac metrics of stress following a yogic breathing workshop: Randomized controlled trial with active comparison. J Am Coll Health, 1-11.

6- Greenberg, N., Docherty, M., Gnanapragasam, S., and Wessely, S. (2020). Managing mental health challenges faced by healthcare workers during covid-19 pandemic. BMJ 368, m1211.

7- Hamilton-West, K., Pellatt- Higgins, T., and Sharief, F. (2019). Evaluation of a Sudarshan Kriya Yoga (SKY) based breath intervention for patients with mild-to-moderate depression and anxiety disorders. Prim Health Care Res Dev 20, e73.

8- Lai, J., Ma, S., Wang, Y., Cai, Z., Hu, J., Wei, N., Wu, J., Du, H., Chen, T., Li, R., et al. (2020). Factors Associated With Mental Health Outcomes Among Health Care Workers Exposed to Coronavirus Disease 2019. JAMA Netw Open 3, e203976.

9- Lovibond, P.F., and Lovibond, S.H. (1995). The structure of negative emotional states: comparison of the Depression Anxiety Stress Scales (DASS) with the Beck Depression and Anxiety Inventories. Behav Res Ther 33, 335-343.

10- Scheier, M.F., Carver, C.S., and Bridges, M.W. (1994). Distinguishing optimism from neuroticism (and trait anxiety, self-mastery, and self-esteem): a reevaluation of the Life Orientation Test. J Pers Soc Psychol 67, 1063-1078.

11- Seppala, E.M., Bradley, C., Moeller, J., Harouni, L., Nandamudi, D., and Brackett, M.A. (2020). Promoting Mental Health and Psychological Thriving in University Students: A Randomized Controlled Trial of Three Well-Being Interventions. Front Psychiatry 11, 590.

12- Trockel, M., Bohman, B., Lesure, E., Hamidi, M.S., Welle, D., Roberts, L., and Shanafelt, T. (2018). A Brief Instrument to Assess Both Burnout and Professional Fulfillment in Physicians: Reliability and Validity, Including Correlation with Self-Reported Medical Errors, in a Sample of Resident and Practicing Physicians. Acad Psychiatry 42, 11-24.

D. COMMITMENT STATEMENT

- The information in the application file is correct.
- The research will be conducted in accordance with the protocol, relevant legislation, Izmir Biomedicine and Genome Center Non-Interventional Ethics Committee Directive, current Helsinki Declaration and Good Clinical Practice principles,
- In case of changes in the research team or changes in the purpose, method, etc. in the research protocol, we will notify your board before the said change is made.
- We confirm that no non-routine procedures such as tests, laboratory examinations, physician examinations, use of chemicals and medical devices, etc. to be performed within the scope of this research; any such procedures will not be covered by public funds and budgets or private health insurances or by the subjects themselves, and that such costs will be paid by the person or organization supporting the project.
- I have read the Good Clinical Practices (GCP) Guideline put into effect by the Republic of Turkey Ministry of Health, I undertake that the above-mentioned study will be conducted in accordance with the principles of this guideline.

Date : 23 / 05 / 2021

Statistical Analyses Plan

All statistical analyses will be performed using Stata, with statistical significance defined as a p-value <0.05.

1) The flow of participants through the study will be represented in a CONSORT diagram.
2) Baseline characteristics and descriptive data of the outcome measures, stratified by the two groups, will be provided using mean and standard deviation, median and interquartile range (IQR) or count and proportion of participants, depending on the data.
3) We will test for significant differences between the groups using t-tests, Wilcoxon Rank Sum test or Chi square/Fisher's exact test, depending on the data.
4) In primary analyses, we will use mixed linear regression models. Separate models will be employed for each outcome measure. The models will include group, timepoint, and an interaction term between group and time (group*time) as fixed effects, and participant's ID as random effect. All models will be tested for normal distribution of the residuals and homoscedasticity. A significant p-value for the time*group interaction will be interpreted as indicative of an intervention effect. The results will also be presented as marginal mean difference with 95%CI between the two groups at the post-tests, and we will illustrate the changes in the outcome measures between the two groups using the marginal means and 95%CI.
5) Additional sensitivity analyses may be performed, and subgroup analyses will be performed to explore a possible impact of adherence to the program.
