# Supplementary material for: Sudarshan Kriya Yoga Breathing and a Meditation Program for Burnout Among Physicians: A Randomized Clinical Trial
Source: JAMA Netw Open. 2024 Jan 31;7(1):e2353978. doi: 10.1001/jamanetworkopen.2023.53978 (PMC10831575; doi:10.1001/jamanetworkopen.2023.53978)
Supplement: Supplement 2. — eTable 1. Mixed Linear Regression Model and High Adherence SKY Group and the Control Group eTable 2. Mixed Linear Regression Model and High and Low Adherence SKY Groups eTable 3. Sensitivity Analyses eFigure 1. DASS-42 Outcomes by Adherence Group and Time Points eFigure 2. LOT-R and RIS Outcomes by Adherence Group and Time Points eFigure 3. PFI Outcomes by Adherence Group and Time Points [file jamanetwopen-e2353978-s002.pdf]

Supplemental Online Content

Korkmaz A, Bernhardsen GP, Cirit B, et al. Sudarshan Kriya Yoga breathing, a meditation-based program, and physician burnout: a randomized clinical trial. *JAMA Netw Open*. 2024;.7(1):e2353978. doi:10.1001/jamanetworkopen.2023.53978

eTable 1. Mixed Linear Regression Model and High Adherence SKY Group and the Control Group

eTable 2. Mixed Linear Regression Model and High and Low Adherence SKY Groups

eTable 3. Sensitivity Analyses

eFigure 1. DASS-42 Outcomes by Adherence Group and Time Points

eFigure 2. LOT-R and RIS Outcomes by Adherence Group and Time Points

eFigure 3. PFI Outcomes by Adherence Group and Time Points

This supplemental material has been provided by the authors to give readers additional information about their work.

eTable1. Mixed Linear Regression Model and High Adherence SKY Group and the Control Group

Marginal mean difference (95%CI) (SKY compared to control group) and p-value from group by time interaction in linear mixed model restricting the SKY group to those

Outcome	High adherence group SKY (n=32)	Control group (n=63)	Marginal mean difference (95%CI)[a]	Group*time *(p-value)*[b]
DASS-42				
Depression,				
Baseline	12.6 (9.0)	14.7 (9.9)		
Post training	6.4 (7.2)	12.5(9.8)**	-6.1 (-9.9, -2.3)	0.010
Post intervention	6.1 (6.7)	11.8 (9.1)**	-5.7 (-9.5, -1.9)	0.020
Anxiety				
Baseline	8.6 (6.0)	10.3 (7.5)		
Post training	4.7 (4.3)	7.6 (7.3)*	-3.0 (-5.8, -0.2)	0.370
Post intervention	4.7 (5.1)	8.1 (7.1)*	-3.4 (-6.2, -0.6)	0.215
Stress				
Baseline	15.6 (7.7)	19.2 (8.9)		
Post training	9.0 (6.9)	16.7 (9.0)***	-7.8 (-11.2, -4.3)	0.009
Post intervention	8.6 (6.5)	15.5 (8.1)***	-6.9 (-10.4, -3.4)	0.037
PFI				
Professional fulfillment				
Baseline	1.94 (0.95)	1.90 (0.97)		
Post training	2.21 (0.99)	1.96 (0.91)	0.24 (-0.16, 0.65)	0.178
Post intervention	2.45 (0.92)	1.98 (1.03)*	0.47 (0.06, 0.88)	0.005
Work exhaustion				
Baseline	2.07 (1.03)	2.19 (1.01)		
Post training	1.51 (0.96)	1.97 (1.09)*	-0.46 (-0.90, -0.02)	0.072
Post intervention	1.26 (0.82)	1.82 (1.14)*	-0.56 (-1.00, -0.13)	0.019
Interpersonal Disengagement				
Baseline	1.45 (0.84)	1.50 (1.00)		
Post training	0.99 (0.69)	1.41 (0.90)*	-0.42 (-0.80, -0.05)	0.058
Post intervention	0.70 (0.67)	1.34 (0.99)**	-0.64(-1.02,-0.26)	0.003
Burnout				
Baseline	1.70 (0.84)	1.78 (0.91)		
Post training	1.20 (0.68)	1.63 (0.91)*	-0.44 (-0.81, -0.07)	0.030
Post intervention	0.92 (0.63)	1.53 (0.98)**	-0.61 (-0.98, -0.24)	0.001
Medical Errors, median (IQR)				
Baseline	2 (3.5)	2 (4)		
Post training	1 (3)	2 (4)	-1.0 (-2.2, -0.2)	0.085
Post intervention	1 (2)	2 (4)	-1.4 (-2.6, -0.1)	0.016
LOT-R				
Baseline	13.0 (3.0)	13.4 (4.2)		
Post training	14.6 (4.1)	13.8 (4.0)	0.8 (-0.9, 2.4)	0.114
Post intervention	15.6 (3.7)	14.1 (4.1)	1.5 (-0.2, 3.1)	0.012
RIS				
Baseline	14.4 (6.1)	13.1 (6.4)		
Post training	13.3 (6.4)	12.2 (5.9)	1.1 (-1.4, 3.7)	0.868
Post intervention	11.6 (4.6)	12.1 (6.5)	-0.4 (-3.0 ,2.1)	0.066

*p<0.05, **p<0.01, ***p<0.001

[a] Marginal mean difference from mixed linear regression analyses, SKY compared to control group.

[b] p-value from interaction term (group*time) from mixed linear regression analyses

DASS - Depression Anxiety and Stress Scale; PFI - Professional Fulfillment and Burnout questionnaire; LOT-R – Revised Life Orientation Test; RIS – Regensburg Insomnia Scale

© 2023 Korkmaz A et al. *JAMA Network Open*.

eTable2. Mixed Linear Regression Model and High and Low Adherence SKY Groups

Marginal mean difference (95%CI), standard deviation (SD) and p-value from group by time interaction in linear mixed model restricting the SKY group to those performing the program ≥3 / week (high adherence group) and ≤2 /week (low adherence group).

Outcome	High adherence group SKY (n=32), Mean (SD)	Low adherence group SKY (n=26), Mean (SD)	Marginal mean difference (95%CI)[a]	Group*time *(p-value)*[b]
DASS-42				
Depression,				
Baseline	12.59 (9.02)	14.61 (9.10)		
Post training	6.37 (7.2)	6.96 (5.28)	-0.59 (-4.45, 3.28)	0.473
Post intervention	6.09 (6.6)	7.54 (7.49)	-1.44 (-5.31, 2.42)	0.773
Anxiety				
Baseline	8.56 (6.05)	8.08 (5..93)		
Post training	4.66 (4.29)	3.23 (2.80)	1.42 (-1.04, 3.90)	0.536
Post intervention	4.66 (5.08)	3.58 (3.91)	1.08 (-1.39, 3.55)	0.696
Stress				
Baseline	15.66 (7.70)	16.12 (8.16)		
Post training	8.97 (6.94)	10.19 (7.04)	-1.22 (-4.96, 2.51)	0.725
Post intervention	8.59 (6.55)	9.92 (7.68)	-1.33 (-5.06, 2.41)	0.689
PFI				
Professional fulfillment				
Baseline	1.94 (0.95)	1.84 (0.72)		
Post training	2.21 (0.99)	2.26 (0.84)	-0.05 (-0.49, 0.39)	0.425
Post intervention	2.45 (0.92)	2.10 (0.75)	0.36 (-0.08, 0.81)	0.137
Work exhaustion				
Baseline	2.07 (1.03)	2.27 (0.97)		
Post training	1.51 (0.96)	1.49 (0.88)	0.02 (-0.46, 0.49)	0.406
Post intervention	1.26 (0.82)	1.75 (0.93)*	-0.49 (-0.97, -0.02)	0.260
Interpersonal Disengagement				
Baseline	1.45 (0.84)	1.36 (0.78)		
Post training	0.99 (0.69)	0.97 (0.72)	0.01 (-0.37, 0.40)	0.695
Post intervention	0.70 (0.67)	1.16 (0.86)*	-0.46 (-0.85, -0.07)	0.006
Burnout				
Baseline	1.70 (0.84)	1.72 (0.78)		
Post training	1.20 (0.68)	1.18 (0.71)	0.02 (-0.36, 0.40)	0.843
Post intervention	0.92 (0.63)	1.40 (0.82)*	-0.47 (-0.85, -0.09)	0.023
Medical Errors, Median (IQR)				
Baseline	2 (3.5)	1.5 (2)		
Post training	1 (3)	2 (3)	0.09 (-1.14, 1.31)	0.341
Post intervention	1 (2)	2 (4)	-0.80 (-2.03, 0.43)	0.018
LOT-R				
Baseline	13.00 (3.05)	13.23 (2.92)		
Post training	14.56 (4.09)	14.00 (4.90)	0.56 (-1.23, 2.36)	0.411
Post intervention	15.56 (3.66)	13.85 (3.38)	1.72 (-0.08, 3.51)	0.044
RIS				
Baseline	14.38 (6.12)	15.04 (5.31)		
Post training	13.34 (6.36)	12.31 (4.93)	1.04 (-1.80, 3.87)	0.124
Post intervention	11.63 (4.60)	12.50 (5.84)	-0.87 (-3.71, 1.96)	0.848

*p<0.05, **p<0.01, ***p<0.001 from t-tests and Mann Whitney U-test

[a] Marginal mean difference from mixed linear regression analyses, high adherence group compared to low adherence group.

[b] p-value from interaction term (group*time) from mixed linear regression analyses

DASS - Depression Anxiety and Stress Scale; PFI - Professional Fulfillment and Burnout questionnaire; LOT-R – Revised Life Orientation Test; RIS – Regensburg Insomnia Scale

© 2023 Korkmaz A et al. *JAMA Network Open.*

eTable 3. Sensitivity Analyses

Sensitivity analyses - Results from mixed linear models with group by time interaction with additional adjustment for sex.

Outcome	Group*time (p-value)
DASS-42	
Depression,	
Baseline	
Post training	0.001
Post intervention	0.001
Anxiety	
Baseline	
Post training	0.117
Post intervention	0.022
Stress	
Baseline	
Post training	0.006
Post intervention	0.028
PFI	
Professional fulfillment	
Baseline	
Post training	0.005
Post intervention	0.002
Work exhaustion	
Baseline	
Post training	0.008
Post intervention	0.038
Interpersonal Disengagement	
Baseline	
Post training	0.033
Post intervention	0.017
Burnout	
Baseline	
Post training	0.007
Post intervention	0.010
Medical Errors, median IQR	
Baseline	
Post training	0.168
Post intervention	0.176
LOT-R	
Baseline	
Post training	0.199
Post intervention	0.089
RIS	
Baseline	
Post training	0.140
Post intervention	0.010

© 2023 Korkmaz A et al. *JAMA Network Open*.

eFigure 1. DASS-42 Outcomes by Adherence Group and Time Points

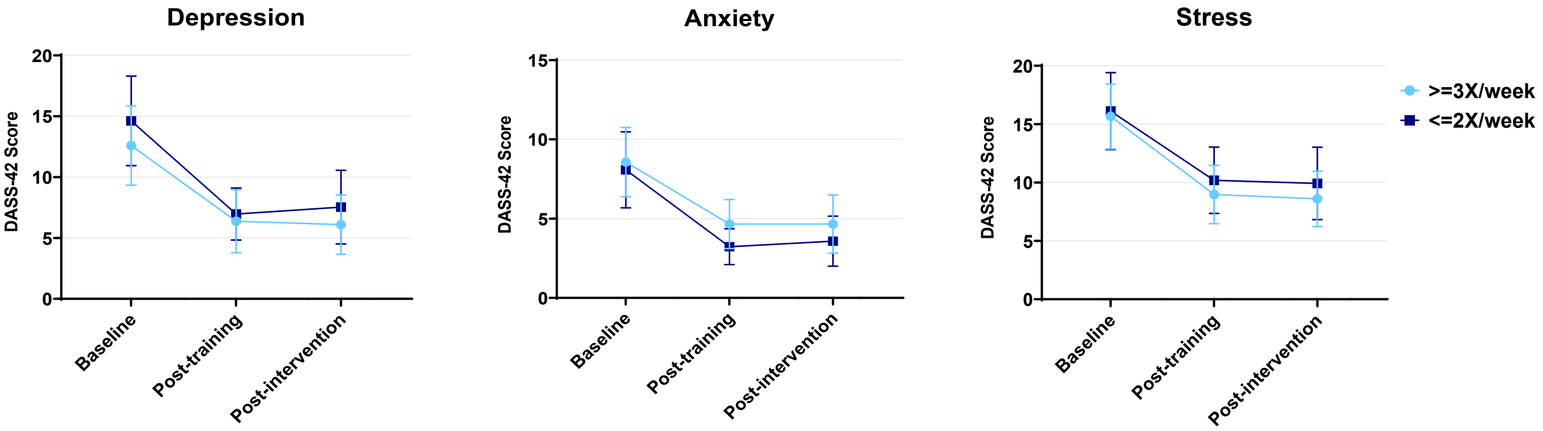

© 2023 Korkmaz A et al. *JAMA Network Open.*

eFigure 2. LOT-R and RIS Outcomes by Adherence Group and Time Points

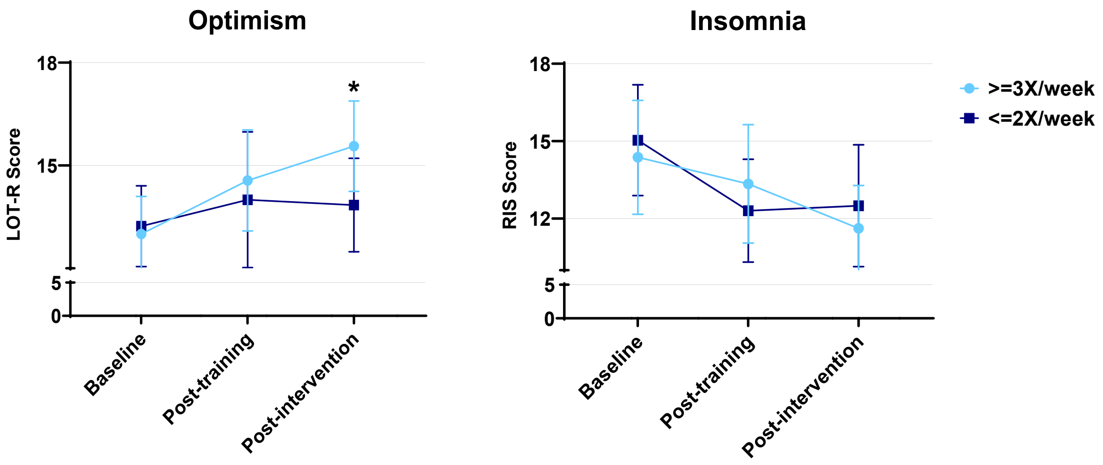

© 2023 Korkmaz A et al. *JAMA Network Open*.

eFigure 3. PFI Outcomes by Adherence Group and Time Points

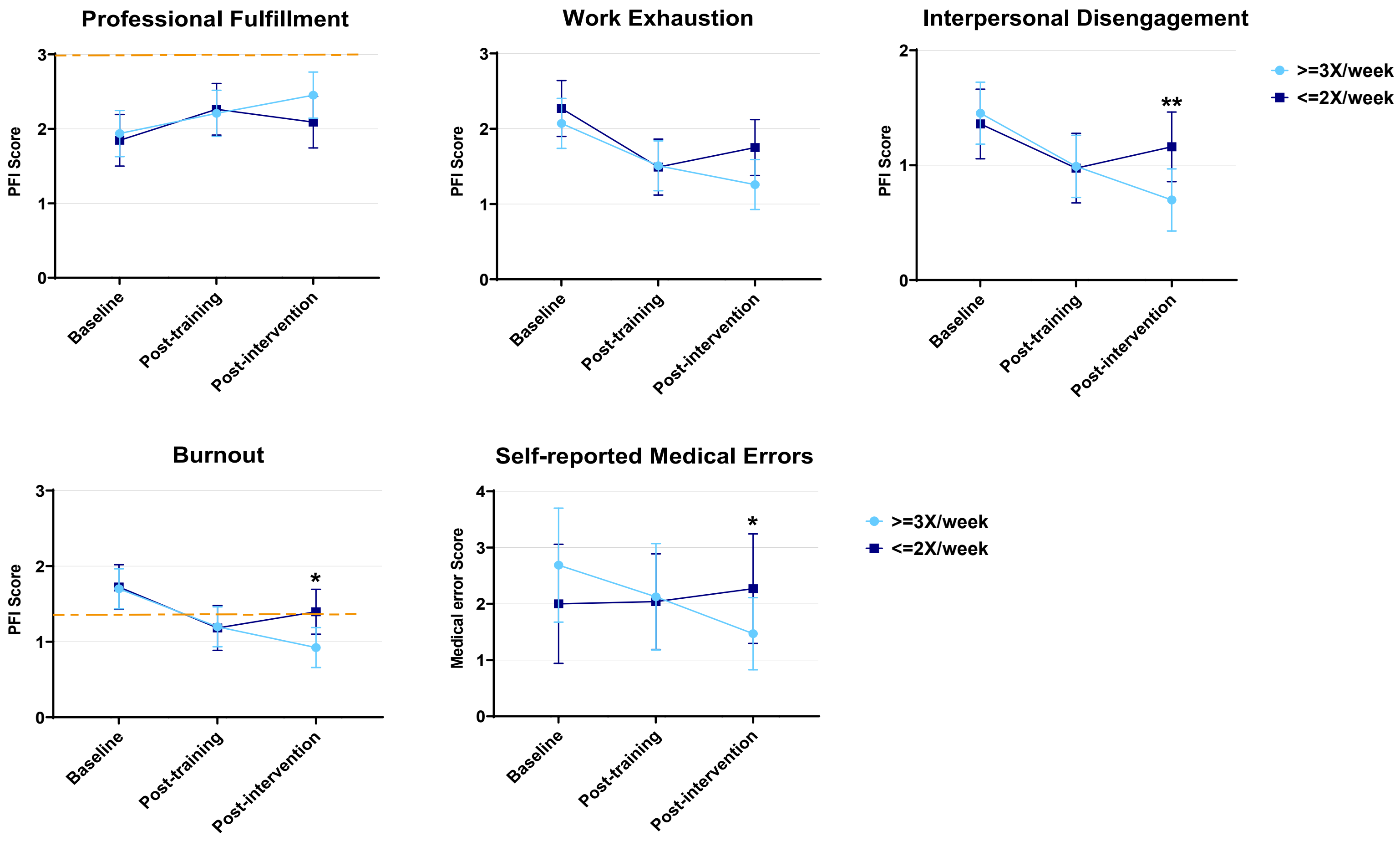

© 2023 Korkmaz A et al. *JAMA Network Open.*
