# Supplementary material for: Sudarshan Kriya Yoga Breathing and a Meditation Program for Burnout Among Physicians: A Randomized Clinical Trial
Source: JAMA Netw Open. 2024 Jan 31;7(1):e2353978. doi: 10.1001/jamanetworkopen.2023.53978 (PMC10831575; doi:10.1001/jamanetworkopen.2023.53978)
Supplement: Supplement 3. — Data Sharing Statement [file jamanetwopen-e2353978-s003.pdf]

Data Sharing Statement

Korkmaz. Sudarshan Kriya Yoga Breathing and a Meditation Program for Burnout Among Physicians. *JAMA Netw Open*. Published January 31, 2024.
doi:10.1001/jamanetworkopen.2023.53978

Data
Data available: No
